# Multi-sectoral data linkages to explore associations between the built environment and BMI.

**DOI:** 10.23889/ijpds.v7i3.2071

**Published:** 2022-08-25

**Authors:** Richard Fry, Lucy Griffiths, Helen Daniels, Amy Mizen, Rhodri Johsnon, Rowena Bailey, James Rafferty, Rebecca Pedrick-Case, Gareth Stratton, Dora Pouliou

**Affiliations:** 1 Swansea University

## Objectives

In Wales almost a quarter of adults and 1 in 8 reception age children are obese. Linked data is a key tool to understanding the role of the built environment on obesity rates and is an important part of developing strategies to combat the obesity epidemic in Wales.

## Approach

We set out to develop an analytical platform for generating evidence on key aspects of the built environment which impact child and adult obesity including; walkability, fast food availability, green space size and qualities, active transport routes and school environments. Utilising the Secure Anonymised Information Linkage (SAIL) Databank We linked multi-sectoral data including routine health data, cohort data, administrative data and linked Geographic Information Systems generated metrics at household and school level. The platform will inform policy makers with and facilitate a better understanding of associations between a range of social, health and built environment factors.

## Results

We have created a range of built environment variables including temporally and age varying walkability indices, viewable greenspace, garden and house size, access to services and parks for 1.5 million households. In the first instance, as part of the BEACHES project, this data has been linked to several health datasets including the Child Measurement Programme (CMP, n=188,800) where initial results have shown that associations between garden size and Body Mass Index in children displays a non-linear negative correlation. We have also created follow-up measures for the CMP using routinely collected general practice data which further enables linking 28,389 height and weight measurements. However, potential bias in these follow-up measures is poorly understood with further work being undertaken to assess usability.

## Conclusion

The integrated multi-sectoral data platform approach to linking environmental, administrative, health and cohort data aims to develop insights on a range of public health issues. We are working with a range of stakeholders to develop evidence-based policy initiatives to reduce obesity in Wales.

